# Molecular dynamics simulation in virus research

**DOI:** 10.3389/fmicb.2012.00258

**Published:** 2012-07-19

**Authors:** Hirotaka Ode, Masaaki Nakashima, Shingo Kitamura, Wataru Sugiura, Hironori Sato

**Affiliations:** ^1^Clinical Research Center, National Hospital Organization Nagoya Medical CenterNagoya, Aichi, Japan; ^2^Pathogen Genomics Center, National Institute of Infectious DiseasesMusashimurayama, Tokyo, Japan; ^3^Department of Biotechnology, Graduate School of Engineering, Nagoya UniversityNagoya, Aichi, Japan; ^4^Department of AIDS Research, Graduate School of Medicine, Nagoya UniversityNagoya, Aichi, Japan

**Keywords:** MD simulation, viral protein, three-dimensional structure, protein dynamics, coarse-grained MD

## Abstract

Virus replication in the host proceeds by chains of interactions between viral and host proteins. The interactions are deeply influenced by host immune molecules and anti-viral compounds, as well as by mutations in viral proteins. To understand how these interactions proceed mechanically and how they are influenced by mutations, one needs to know the structures and dynamics of the proteins. Molecular dynamics (MD) simulation is a powerful computational method for delineating motions of proteins at an atomic-scale via theoretical and empirical principles in physical chemistry. Recent advances in the hardware and software for biomolecular simulation have rapidly improved the precision and performance of this technique. Consequently, MD simulation is quickly extending the range of applications in biology, helping to reveal unique features of protein structures that would be hard to obtain by experimental methods alone. In this review, we summarize the recent advances in MD simulations in the study of virus–host interactions and evolution, and present future perspectives on this technique.

## Introduction

**Figure 1:**
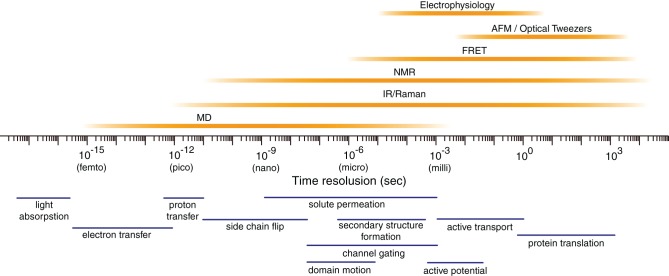
**Temporal resolution of various biophysical techniques.** The timescales of some fundamental atom- or molecule-scale motions are shown below. AFM, atomic force microscopy; FRET, fluorescence resonance energy transfer; IR, infrared radiation; NMR, nuclear magnetic resonance.

Proteins fluctuate spontaneously in solution (Ishima and Torchia, 2000). Accumulating evidence indicates that such fluctuations play key roles in the specific functions of proteins, such as catalytic reactions of enzymes (Nicholson et al., 1995; Lu et al., 1998; Eisenmesser et al., 2005; Henzler-Wildman et al., 2007; Abbondanzieri et al., 2008), interactions with other biomolecules (Thorpe and Brooks, 2007), and biomolecular motors and pumps (Astumian, 1997). Multiple experimental methods are available to characterize the protein dynamics (Figure 1). However, it is usually difficult to delineate motions of proteins at an atomic scale.

## MD simulation in biology

### Outline

Molecular dynamics (MD) simulation is a computational method to address the above issue (Figure 1) (Henzler-Wildman and Kern, 2007; Dror et al., 2010). This technique enables us to calculate movements of atoms in a molecular system, such as proteins in water, by numerically solving Newton's equations of motions (Karplus and Petsko, 1990; Adcock and McCammon, 2006). In a simple molecular system, all atoms and covalent bonds connecting the atoms are assumed to be the charged spheres and springs, respectively. Parameters of mathematical functions describing the potential energy of a system, termed the “force field,” are set to simulate the movements of atoms and molecules. Frequently used force fields for proteins, such as the “AMBER” (Pearlman et al., 1995; Case et al., 2005) and “CHARMM” (Brooks et al., 2009) force fields, have the formulae of covalent bonds, angles, dihedrals, van der Waals, and electrostatic potentials.

### Performance and consistency with experimental data

Application of MD simulation in the field of protein chemistry was first reported in 1977 (McCammon et al., 1977). Since then, the performance of this technique have been quickly improved quantitatively and qualitatively along with the rapid advances in hardware and software on biomolecular simulation (Lindorff-Larsen et al., 2012). The results of MD simulation are critically influenced by the force fields (Lindorff-Larsen et al., 2012). The qualities of parameters in the force fields, especially for dihedrals and electrostatic potentials, have been improved quantitatively and qualitatively over time by introducing improved approximation to the quantum ground-state potential energy surface. Recently, eight different protein force fields were evaluated on the basis of the consistency of simulations with the NMR data (Lindorff-Larsen et al., 2012). The study demonstrates that the most recent versions, while not perfect, provide results that are highly consistent with the experimental data (Lindorff-Larsen et al., 2012). In addition, explicit introduction of effects of the solvation has contributed to the qualitative improvement for the precision and performance of MD simulations (Adcock and McCammon, 2006).

### MD in structural biology

MD simulation currently allows us to investigate the structural dynamics of proteins on timescales of nanoseconds to microseconds, and will probably allow investigation to milliseconds in the future (Figure 1) (Henzler-Wildman and Kern, 2007; Dror et al., 2010). This technique is widely used in the field of structural biology (Karplus and McCammon, 2002; Karplus and Kuriyan, 2005; Dodson et al., 2008). First, MD simulation is useful for refining the experimentally determined three-dimensional (3-D) structures of proteins (Autore et al., 2010; Ozen et al., 2011). Second, MD simulation is beneficial for constructing previously undescribed 3-D structures of proteins in combination with homology modeling techniques (Marti-Renom et al., 2000; Sanchez et al., 2000; Baker and Sali, 2001), when a reported structure of a homolog is available. Third and most importantly, MD simulation provides a unique tool to address the structural dynamics of proteins, i.e., the time evolution of conformations in solution, at timescales of nanoseconds to microseconds (Henzler-Wildman and Kern, 2007; Dror et al., 2010). The structural snapshots obtained during MD simulation are helpful for depicting the unique structural features of proteins (Karplus and McCammon, 2002; Karplus and Kuriyan, 2005; Dodson et al., 2008).

## MD simulation in virology

To date, MD simulations have been applied in a range of virus researches, as shown in the following sections.

### Neutralization escape and cell tropism switching of HIV-1 mediated by an electrostatic mechanism

It is very important to clarify how viruses evade neutralization antibodies in order to understand the viral life cycle and evolution, and to develop vaccines. MD simulation is used to address this issue as it pertains to human immunodeficiency virus type 1 (HIV-1). The third variable (V3) loop of the HIV-1 envelope gp120 protein constitutes the major antibody epitopes of HIV-1 and the major determinants for the entry coreceptor use of HIV-1. By analyzing the 40,000 structural snapshots obtained from 10–30 ns of MD simulations of the identical gp120 outer domain carrying a distinct V3 loop with net charge of +3 or +7, Yokoyama and colleagues showed that the change in V3 net charge alone is sufficient to induce global changes in fluctuation and conformation of the loops involved in binding to CD4, coreceptor, and neutralizing antibodies (Naganawa et al., 2008; Yokoyama et al., 2012). Structural changes caused by a reduction in the V3 net charge via V3 mutations are tightly linked to viral CCR5 coreceptor tropism (Naganawa et al., 2008), as well as to a reduction in viral neutralization sensitivity to anti-V3 antibodies (Naganawa et al., 2008) and anti-CD4 binding site monoclonal antibodies (Yokoyama et al., 2012). These findings suggest a hitherto unrecognized mechanism, V3-mediated electrostatic modulation of the structure and dynamics of the gp120 interaction surface, for adjusting the relative replication fitness and evolution of HIV-1 (Yokoyama et al., 2012). In addition, they partly explain a virological mystery, i.e., why HIV-1 variants using CCR5, which carries a V3 loop with a lower level of positive net charge, predominantly persist before the onset of AIDS.

### Mechanisms of viral escape from host defense systems

**Figure 2:**
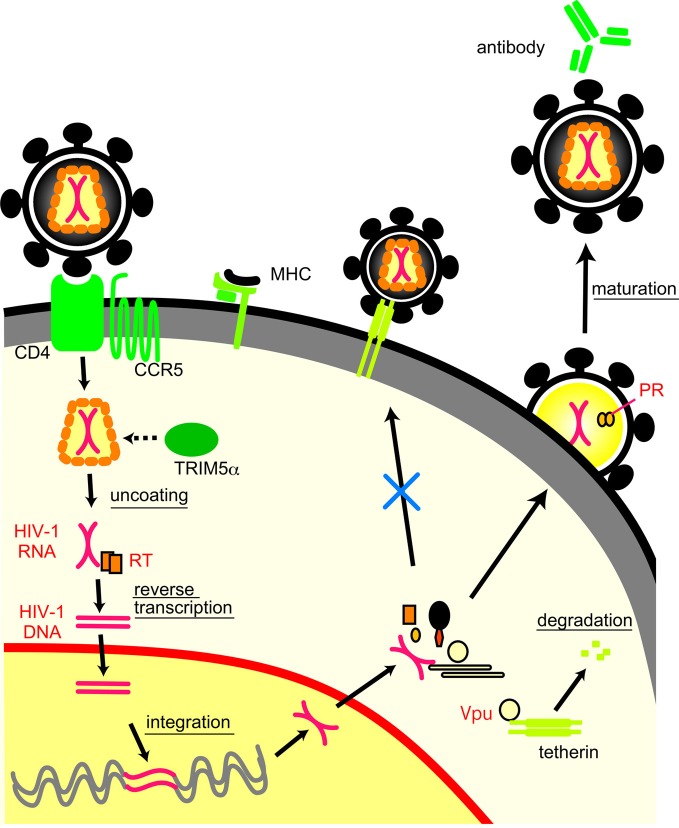
**Life cycle of HIV-1 and interactions between viral proteins and host immune molecules**.

Viruses also evade host defense systems other than neutralization antibodies (Figure 2). MD simulation is used to clarify the structural basis for viral escape from host defense systems by mutations. Mutations at the 120th amino acid in the HIV-2 capsid protein play a key role in evading tripartite motif-containing protein 5α (TRIM5α), an anti-retroviral cellular protein induced by interferon, both *in vivo* (Onyango et al., 2010) and *in vitro* (Song et al., 2007). An MD simulation study has revealed that the mutations could extensively influence the conformation and fluctuation of the interaction surface of capsid proteins by altering the probability of hydrogen bond formation between helices 4 and 5 (Miyamoto et al., 2011).

HIV-1 Vpu antagonizes an antiviral cellular protein termed tetherin, also known as BST-2/CD317/HM1.24, by interaction with the transmembrane (TM) domain of tetherin and subsequent degradation (Douglas et al., 2010; Kobayashi et al., 2011). An MD simulation suggests that alignment of the four amino acid residues (I34, L37, L41, and T45) on the same helical face in the human tetherin TM domain is crucial for the Vpu-mediated antagonism against human tetherin (Kobayashi et al., 2011). The interface structure of the tetherin TM for the antagonism was also predicted by the MD simulation of another group (Zhou et al., 2012) and experimentally confirmed by an NMR study (Skasko et al., 2012).

MD simulation is also used to study the mechanisms of functional interactions between cytotoxic T lymphocyte (CTL) epitope and major histocompatibility complex (MHC) molecules (Reboul et al., 2012). An MD simulation study has revealed that a 13-mer epitope peptide from Epstein-Barr virus has the low structural flexibility in an MHC molecule that induces a CTL response but exhibits high flexibility in another MHC molecule that cannot induce a CTL response (Reboul et al., 2012). Thus, structural flexibility of CTL epitope region seems to be critical for the specific recognition by MHC molecules, and mutations that alter the flexibility may influence CTL response. There are other viral proteins and immune molecules involved in viral evasion from host defense systems (Neil and Bieniasz, 2009; Malim and Bieniasz, 2012). MD simulations should also be applicable for the studies of these molecules.

### Structure and function of viral enzymes

Viral enzymes are essential for viral replications and thus are important targets for anti-viral drug development. MD simulations are used to study the basis of the structural dynamics that allow the viral enzyme and its drug to function properly. Viral polymerase (Pol) is essential for viral genome replication in the viral life cycle. The Pol is composed of the fingers, palm, and thumb subdomains, which form a cavity for the substrate binding, as in eukaryotic Pol (Joyce and Steitz, 1994; Lamers et al., 2006; Cameron et al., 2009). MD simulations suggest that the finger and thumb domains of HIV-1 reverse transcriptase (RT) are especially mobile among the various regions of this enzyme (Zhou et al., 2005; Kirmizialtin et al., 2012). The mobility is severely attenuated by binding of allosteric non-nucleotide RT inhibitors (NNRTIs) (Zhou et al., 2005). Interestingly, a large conformational change of RT subdomains during millisecond timescale simulations can lock the correct nucleotide at the active site but promotes release of a mismatched nucleotide (Kirmizialtin et al., 2012). Furthermore, conformational dynamics leading to opening and closing motions of the substrate binding cleft are highly conserved among four RNA Pols in the picornavirus family, despite the amino acid identity being as low as 30–74% (Moustafa et al., 2011). These findings are consistent with each other and strongly suggest that the structural dynamics of viral Pol play a key role in the polymerization.

Viral protease (PR) plays a key role in viral propagation by catalyzing cleavages of viral precursor proteins (Pettit et al., 1994, 2002; Steven et al., 2005). HIV-1 PR and other retroviral PRs have unique regions termed the “flaps” outside the substrate binding clefts (Dunn et al., 2002). MD simulation studies suggest that the PR flaps in HIV-1 are intrinsically mobile, undergoing conversions between the “semiopen,” “open,” and “closed” conformations (Hornak et al., 2006; Deng et al., 2011). This movement is severely attenuated upon placement of the substrate or PR inhibitor in the binding cleft (Karthik and Senapati, 2011), suggesting that flap movement plays a critical role in PR function.

MD simulations are also used to study the structural dynamics of the substrates of viral PR. Peptides corresponding to cleavage junctions of viral precursor proteins of HIV-1 are intrinsically unstructured in aqueous solution (Datta et al., 2011; Ode et al., 2011). However, the folding preference of the junction peptides may be different among the junctions and related to the efficiency of substrate binding and cleavage reaction by PR (Ode et al., 2011). Furthermore, peptides at the capsid-p2 junction can adopt a helical conformation when the polarity of the environment is reduced (Datta et al., 2011). The MD simulation of PR and its substrates will help to clarify how the viral precursor is processed orderly during viral maturation.

### Drug-resistance mechanisms

Antiviral drug resistance is a major clinical problem for the treatment of virus-infected individuals (Cortez and Maldarelli, 2011; van der Vries et al., 2011). Viral resistance to antiviral drugs is primarily caused by genetic mutations that eventually lead to a reduction in the drug affinity of drug target viral proteins. MD simulations are used to examine how viral mutations cause the drug resistance at the atomic level.

A reduction in the binding affinity of the PR inhibitors to HIV-1 PR can be caused by a reduction in hydrophobic interactions (Kagan et al., 2005; Wittayanarakul et al., 2005; Sadiq et al., 2007; Chen et al., 2010; Dirauf et al., 2010), reduction in electrostatic interactions (Ode et al., 2005, 2006, 2007a; Chen et al., 2010), changes in flexibility at the flap of the PR (Piana et al., 2002; Perryman et al., 2004; Chang et al., 2006; Foulkes-Murzycki et al., 2007), and changes in the shape of the inhibitor-binding pocket (Ode et al., 2005, 2006, 2007b). Reduction in binding affinity of the nucleotide/nucleoside RT inhibitors (NRTIs) to HIV-1 RT can be caused by a distinct conformational preference of NRTIs in the substrate/NRTI-binding site compared to normal substrates (Carvalho et al., 2006) or enhancement of ATP-mediated excision of misincorporated nucleotide analogs via increased accessibility of ATP to the terminus of extending DNA (White et al., 2004; Carvalho et al., 2007). Reduction in the binding affinity of the NNRTIs to HIV-1 RT can be attained by occlusion of the NNRTI-entry pathway (Rodriguez-Barrios and Gago, 2004; Rodriguez-Barrios et al., 2005) or restoration of the proper flexibility of the RT even with NNRTIs (Zhou et al., 2005).

A change in volume of the binding site of influenza virus (IFV) M2 channel blockers has been shown to reduce the blockers' binding affinity (Gu et al., 2011; Leonov et al., 2011; Wang et al., 2011). Disruption of the proper guidance of IFV neuraminidase (NA) inhibitors into their binding pocket is proposed as a possible mechanism for the reduction in the binding affinity of the inhibitors (Le et al., 2010; Kasson, 2012). MD simulations are also used to study how the genetic differences of HIV variants around the world can influence the efficacy of antiviral inhibitors (Batista et al., 2006; Ode et al., 2007a; Matsuyama et al., 2010; Soares et al., 2010; Kar and Knecht, 2012). Thus, MD simulation will be valuable to assist in the study of drug efficacy when genetic information on the drug target proteins is available (Shenderovich et al., 2003; Stoica et al., 2008; Sadiq et al., 2010; Wright and Coveney, 2011).

### Antiviral drug discovery and development

MD simulations are used to assist in the discovery and development of antiviral drugs (Durrant and McCammon, 2011; Borhani and Shaw, 2012). MD simulations allow sampling snapshots of fluctuated protein structures, which include their short-lived conformations as well as stable conformations. This is beneficial for searching conformations of a protein on ligand-binding, since ligand-binding can stabilize conformation of a protein that is not the most stable at ligand-free state (Tobi and Bahar, 2005; Xu et al., 2008). Thus, the MD simulations are used to improve the enrichment performance of molecular docking during *in silico* drug screening by taking accounts of multiple docking poses (Okimoto et al., 2009). The method is also applied for identifying concealed drug-binding sites, which are apparently masked and not evident from the X-ray crystal structures, by considering the structural flexibility of proteins. For example, MD simulations have been used to find a trench adjacent to the active site of HIV-1 integrase (Schames et al., 2004). A site-directed mutagenesis study provided evidence that the trench indeed plays key roles in ligand-binding (Lee and Robinson, 2006). These findings have been used to design HIV-1 integrase inhibitors with potent antiviral effects (Durrant and McCammon, 2011).

Likewise, MD simulations are used to assist in the development of antiviral drugs against IFV. Using this method, a universal cavity adjacent to the binding site of natural substrate has been reported with NA proteins of human 2009 pandemic H1N1, avian H5N1, and human H2N2 strains (Amaro et al., 2011). MD simulations were also used to construct a 3-D structure model of CCR5, a major coreceptor of HIV-1 (Maeda et al., 2008; Da and Wu, 2011).

### Virion structure

**Figure 3:**
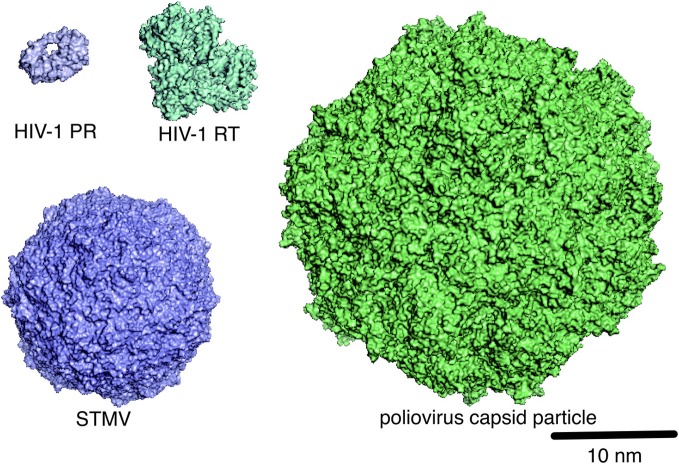
**Molecular scales of viral proteins and capsid particles.** The structures of HIV-1 PR (PDB code: 1HHP), HIV-1 RT (PDB code: 1RTD), STMV (PDB code: 1A34), and poliovirus capsid particle (PDB code: 1HXS), which are deposited in the Protein Data Bank (PDB) (http://www.rcsb.org/pdb/home/home.do) or the VIPERdb (http://viperdb.scripps.edu/), are shown in surface representation.

It is essential to clarify the structure of virions in order to understand the mechanisms of viral infection and assembly. MD simulation is used to address this issue. Using a super computer, Freddolino et al. performed 50-nanosecond-timescale MD simulations of the symmetric structure of a complete satellite tobacco mosaic virus (STMV) particle containing about 1 million atoms (Arkhipov et al., 2006) (Figure 3). Thus, far, this is one of the largest systems among the MD simulations reported in all biological fields. Notably, the virion with viral RNA was stable during the simulations, whereas the one without the RNA was unstable, suggesting that viral RNA plays a key role in stabilizing the STMV virion (Arkhipov et al., 2006). The study is consistent with the experimental data (Day et al., 2001) and therefore provides a set of rationale conditions for performing the MD simulation of virion. Likewise, Larsson et al. reported about 1-microsecond-timescale MD simulations of the satellite tobacco necrosis virus (STNV) (Larsson et al., 2012). Their study reproduced the biochemical phenomenon of the STNV virion in solution (Unge et al., 1986), i.e., the swelling of capsid upon Ca^2+^ removal by EDTA treatment. These findings will provide a structural basis for identifying the key regulators of assembly and infections and for illustrating how they function mechanically. Although MD simulation of virions composed of very large numbers of atoms is still difficult in most cases, progress in the hardware and software for the simulation, together with the accumulation of biological and physicochemical information on virions, will help us to overcome these limitations in the MD simulation of virions.

## Perspective

Since the processing speed of computers is still doubling approximately every two years according to Moore's law, MD studies will be extended to simulations of larger and more complex system at longer timescales. This will then lead to a better understanding of the structures and dynamics of macromolecules involved in virus–host interactions.

### Coarse-Grained (CG) MD simulations

MD simulations of macromolecules consisting of large molecular systems, such as oligomeric proteins, macromolecular complexes, and membrane proteins in a lipid bilayer, and virions are desired to better understand viruses. However, such simulations require unrealistically long analytical times and high-performance computers at present, and thereby are still limited mostly to the small molecules (Henzler-Wildman and Kern, 2007; Dror et al., 2010). To cope with this issue and to improve the practicability of long timescale MD simulation, a “coarse-grained (CG) MD” simulation has been developed (Merchant and Madura, 2011; Takada, 2012). The CG-MD simulation employs “pseudo-atoms” that consist of several atoms in a group and calculates the movement of these “pseudo-atoms” rather than the movement of “individual atoms,” thereby greatly reducing the calculation time (Merchant and Madura, 2011; Takada, 2012). CG-MD simulations have been used to study helicases of hepatitis C virus (HCV) and simian virus 40 and have successfully reproduced enzyme motions, such as “ratcheting inchworm translocation” and “spring-loaded DNA unwinding” (Flechsig and Mikhailov, 2010; Yoshimoto et al., 2010). Briefly, the ratcheting inchworm translocation is the unidirectional motion of the HCV NS3 helicase during translocation that occurs by the step size of one base per ATP hydrolysis cycle (Gu and Rice, 2010). Meanwhile, the spring-loaded DNA unwinding is the discrete steps of unwinding of DNA by the HCV NS3 helicase that occurs periodically via a burst of 3-bp unwinding during NS3 translocation consuming ATPs (Myong et al., 2007).

CG-MD has also been applied to the study of the structural characteristics and stabilities of the capsid particle and virion (Figure 3). Such studies have been used to investigate small plant viruses (~28 nanometer in diameter), such as the three satellite plant viruses STMV, STNV, and the satellite panicum mosaic virus (SPMV), as well as the brome mosaic virus (BMV) (Arkhipov et al., 2006, 2009), and more complex capsids such as poliovirus (Arkhipov et al., 2006, 2009), asymmetric, conical-shaped HIV-1 capsid particles (Krishna et al., 2010), and the immature HIV-1 virion (Ayton and Voth, 2010). These studies have predicted various molecular interactions that can be tested experimentally. Thus, CG-MD may play a pivotal role in the MD study of micrometer-sized systems at millisecond timescale (Merchant and Madura, 2011; Takada, 2012) and therefore may uncover novel characteristics of the interactions in virus–host relationships.

### Intrinsically disordered proteins

Some eukaryotic proteins have no stable 3-D structure under physiological conditions (Dunker et al., 2002, 2008; Dyson and Wright, 2005). These proteins are referred to as intrinsically disordered, natively unfolded, or intrinsically unstructured proteins. They undergo structural transition from a disordered to an ordered state upon binding to target molecules such as proteins, DNA, and small molecules (Dunker et al., 2005; Sandhu and Dash, 2007). They are often related to the “hub proteins” that have many binding partners and control important biological processes (Iakoucheva et al., 2002; Haynes et al., 2006; Sandhu, 2009). Interestingly, viral proteins or portions of viral proteins are often intrinsically disordered. These include genome-linked protein VPg protein of plant viruses (Grzela et al., 2008; Rantalainen et al., 2008; Jiang and Laliberte, 2011; Rantalainen et al., 2011), HIV-1 Tat (Shojania and O'Neil, 2010), and Vif proteins (Reingewertz et al., 2010), and paramyxovirus nucleoproteins and phosphoproteins (Habchi and Longhi, 2012). It has been proposed that the disordered structure is beneficial for viruses to gain multiple functions in the viral life cycle with limited genome size (Rantalainen et al., 2011; Habchi and Longhi, 2012; Xue et al., 2012). Clarifying the folding landscape of viral proteins by standard MD and CG-MD simulations may help in understanding the structural principles by which viral proteins execute multiple functions in the viral life cycle.

### Conflict of interest statement

The authors declare that the research was conducted in the absence of any commercial or financial relationships that could be construed as a potential conflict of interest.
